# Growth dynamic of biofilm-associated *Naegleria fowleri* in freshwater on various materials

**DOI:** 10.3389/fmicb.2024.1369665

**Published:** 2024-03-06

**Authors:** Sébastien Goudot, Laurence Mathieu, Pascaline Herbelin, Sylvie Soreau, Frédéric P. A. Jorand

**Affiliations:** ^1^EDF Recherche et Développement, Laboratoire National d'Hydraulique et Environnement, Chatou, France; ^2^Université de Lorraine, CNRS, LCPME, Nancy, France; ^3^EPHE, PSL, UMR CNRS 7564, LCPME, Nancy, France

**Keywords:** *Naegleria fowleri*, brass, stainless steel, PVC, titanium, freshwater biofilm, free-living amoeba

## Abstract

In industrial water systems, the occurrence of biofilm-associated pathogenic free-living amoebae (FLA) such as *Naegleria fowleri* is a potential hygienic problem, and factors associated with its occurrence remain poorly understood. This study aimed to evaluate the impact of four cooling circuit materials on the growth of *N. fowleri* in a freshwater biofilm formed at 42°C and under a hydrodynamic shear rate of 17 s^−1^ (laminar flow): polyvinyl chloride, stainless steel, brass, and titanium. Colonization of the freshwater biofilms by *N. fowleri* was found to be effective on polyvinyl chloride, stainless steel, and titanium. For these three materials, the ratio of (bacterial prey)/(amoeba) was found to control the growth of *N. fowleri*. All materials taken together, a maximum specific growth rate of 0.18 ± 0.07 h^−1^ was associated with a generation time of ~4 h. In contrast, no significant colonization of *N. fowleri* was found on brass. Therefore, the contribution of copper is strongly suspected.

## 1 Introduction

The use of freshwater for industrial processes, such as cooling water systems or drinking water networks, provides a favorable aquatic environment for the growth of highly diverse and ubiquitous microorganisms. From recent studies, it appears that biofilms readily grow on the extensive and diverse surfaces present in industrial water systems, such as heat exchangers, cooling towers, or water tanks. These biofilms are preferred ecological reservoirs for free-living amoebae (FLA), where they crawl to find nutrients (bacterial cells and dissolved organics) (Hunt and Parry, 1998; Barbeau and Buhler, 2001; Parry, 2004; Huws et al., 2005; Loret et al., 2005; Puzon et al., 2009, 2017; Taravaud et al., 2018; Fritz-Laylin, 2020).

Among the large variety of FLA species present in these ecosystems, *Naegleria fowleri*, a thermotolerant amoeboflagellate of health-related interest, has been isolated as the causative agent of primary amoebic meningoencephalitis, a fatal central nervous system disease (Marciano-Cabral, 1988; Gharpure et al., 2021; Zhang and Cheng, 2021; Kofman and Guarner, 2022). *Naegleria fowleri* can be frequently encountered in thermally enriched cooling water (Behets et al., 2007; Jamerson et al., 2009; Lam et al., 2019; Logan-Jackson and Rose, 2021).

Although the occurrence of this pathogenic FLA has been reported, only a few studies have attempted to identify the factors that promote its occurrence, survival and growth. Only temperature, excluding low salinity conditions and also biofilms has been reported as a key parameter (De Jonckheere and Voorde, 1977; Wellings et al., 1977; Brown et al., 1983; Tyndall et al., 1989; Huizinga and McLaughlin, 1990; Jamerson et al., 2009; Goudot et al., 2012; Lam et al., 2019; Shaheen et al., 2019; Del Olmo et al., 2021; Stahl and Olson, 2021; Hoque et al., 2023).

However, many other factors could also influence FLA, including *N. fowleri*, to colonize environments: bacterial/amoeba ratio (BAR), chemicals (e.g., disinfectant residual), organic carbon, interactions with other eukaryotes, or the nature of the substrata, the material colonized by the biofilm. The effect of this substrata on biofilm-associated microorganisms has been widely studied (Niquette et al., 2000; Batté et al., 2003; Waines et al., 2011; Liu et al., 2014; Pinto et al., 2019; Douterelo et al., 2020; Gomes et al., 2022), but its consequence on the FLA has remained poorly documented. The FLA could be indirectly impacted by the bacterial diversity and cell density changes or directly by the chemicals associated with the material.

Therefore, the objective of this work was to determine whether the nature of support materials, such as those encountered in cooling systems, can represent a major influencing factor in the development of *N. fowleri*. In this context, the present study aims to (i) evaluate the growth dynamics of biofilm-associated *N. fowleri* in freshwater biofilms and (ii) compare the behavior of *N. fowleri* within the biofilm formed on four cooling waters extensively used materials: polyvinyl chloride, stainless steel, brass, and titanium. The assays were conducted on a biofilm reactor, which allowed freshwater biofilms to develop from raw river waters and experimentally introduced pathogenic amoebae to colonize at 42°C for 45 days. In addition, the colonization of glass material was monitored under the same conditions to serve as a reference material.

## 2 Materials and methods

### 2.1 *Naegleria fowleri* strain, culture conditions and inoculum preparation

The AMI005 strain of *N. fowleri* (EDF internal collection, LNHE, Chatou, France) was isolated from the cooling water of a power station (unpublished data). *N. fowleri* was grown (for 3–5 days) at 43°C on non-nutrient agar (NNA, Indicia Biotechnology, Oullins, France) previously overlaid with an *Escherichia coli* suspension and identified by an enzyme-linked immunosorbent assay (Indicia Biotechnology) using monoclonal antibody 5D12 (Pougnard et al., 2002). The maintenance of the strain was carried out by transplanting every 3 months. Subculturing consists of cutting a cube of agar and placing it with the contaminated side on a new medium. Incubation was then carried out between 2 and 5 days at 43°C. Following this maintenance procedure, the strain was kept in the dark at 20–25°C in the culture box (sealed tightly with parafilm). *N. fowleri* trophozoites were harvested under microscope examination after a 2-day culture on *E. coli* mats against a 5-day culture for cysts. Suspensions of *N. fowleri* trophozoites were prepared by gently scraping the amoebic migration front of 10 plates and poured them into 5 ml of phosphate buffer saline solution at a pH value of 7.4 (PBS) for further use.

### 2.2 Biofilm bacterial cell counting and physicochemical analysis

The biofilm on the coupons was first removed by scraping with a sterile swab into 150 ml of bacteria-free PBS. Consequently, the swab containing the extracted biofilm and the coupons were treated with ultrasounds for 10 min (ultrasonic bath, 140 W, 50/60 Hz; Fisher Scientific). As recently reviewed by Azeredo et al. (2017), the methodologies used for biofilm detachment are of great importance; therefore, we have preliminary verified and optimized the power and duration of the ultrasound treatment with respect to the highest cell detachment and lowest mortality of amoebae (Goudot et al., 2012; Perrin et al., 2018). Bacterial and amoebic analyses were conducted on the same dispersed biofilm from the same coupon.

The number of bacterial cells in the biofilm extracts was determined by direct count using epifluorescence microscopy. The cells were stained with the DNA fluorochrome SybrGreen I (S7567, InVitrogen) and recovered on a black nucleopore filter with a membrane of 0.2 μm pore size (Goudot et al., 2012).

Dry weight and copper analysis have been performed on biofilm extracted from some campaigns. Copper was analyzed by anodic stripping voltammetry and the standard addition method after mineralization. The detection limit was 0.1 μg mg^−1^ dry weight.

### 2.3 *Naegleria fowleri* and thermophilic FLA cell counting

Thermophilic FLA, including *N. fowleri*, were counted using the most probable number (MPN) approach described by Pougnard et al. (2002). The MPN approach determines the concentration of viable *N. fowleri*—both trophozoites and cysts—without allowing the two forms to be discriminated. Briefly, five replicates of 1 ml of samples (with an appropriate dilution) were spread onto NNA plates previously inoculated with *E. coli*. The plates were incubated at 43°C, and the presence of an amoebic migration front was assessed daily for 5 days by microscopic examination. Amoebae fronts were further analyzed to determine the presence of *Naegleria* species using a flagellation test by incubating vegetative forms in demineralized water at 37°C for 4 h (Behets et al., 2003). Among the flagellated amoebae, *N. fowleri* were identified using an enzyme-linked immunosorbent assay (Indicia Biotechnology) with monoclonal antibody 5D12 as previously described by Reveiller et al. (2003). All other detected amoebae that do not meet flagellation and ELISA tests were classified as thermophilic FLAs other than *N. fowleri*.

### 2.4 Biofilm formation and setup of *Naegleria fowleri* inoculation

**Figure 1:**
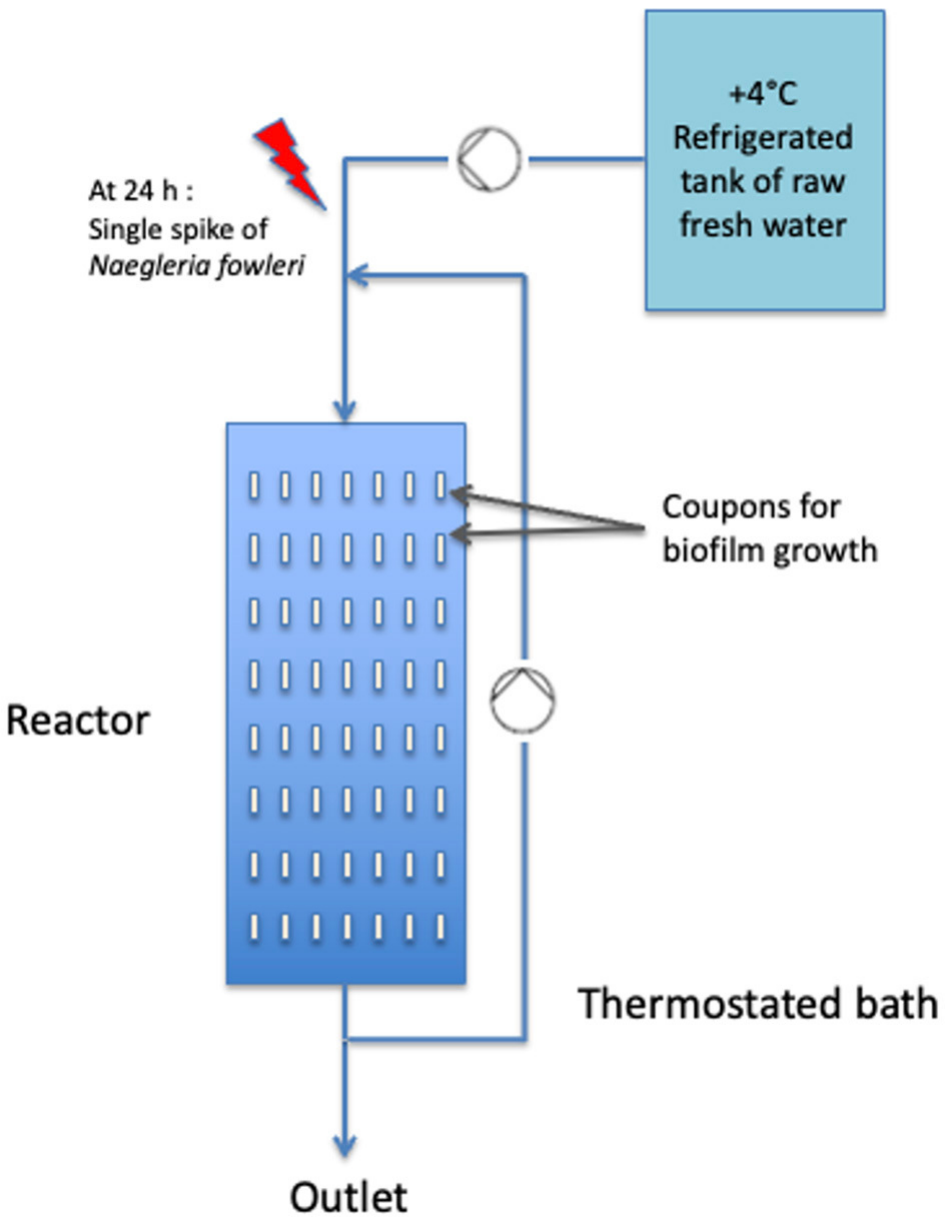
Schematic view of the experimental setup. For a more detailed scheme see Goudot et al. (2012).

A flat-plate open-channel reactor previously described by Goudot et al. (2012) was operated in continuous flow mode. The inlet flow and the recycle flow rates were maintained at 1.9 and 810 ml min^−1^, respectively. The hydraulic retention time was 24 h. The flow presented a laminar velocity profile in the length direction, characterized by a shear rate of 17 s^−1^. The bacteria present in the river water adhered spontaneously to the different surfaces of the reactor. The biofilm grew at 42°C on slide coupons measuring ~22 cm^2^ (8 × 2.5 × 0.1 cm) at the bottom of the reactor (Goudot et al., 2012) made of different test materials (polyvinyl chloride, stainless steel, brass, and titanium). From 24 h on, it is expected that the biofilm will present ~10^6^ bacteria cm^−2^. A schematic view of this experimental setup is presented in Figure 1.

Test campaigns (named C1–C6) were conducted for a period of 45 days each. Each test campaign was conducted with the reactor fed by the same fresh water. For C1–C4, two runs (named R1 and R2) were consecutively performed; for C5 and C6, a single run was performed (R1). The assignments for each independent campaign are shown in Table 1. Between each run, the reactor was acid-cleaned (HCl 1 M, 4 h), disinfected (100 mg Cl_2_ L^−1^, 4 h), and finally rinsed with deionized water (five times the working volume).

**Table 1:** Assignments of assays for the six campaigns.

**Test campaigns**	**Assignments of the assays**	
	**Run 1 (R1)**	**Run 2 (R2)**
Campaign No. 1 (C1)	Polyvinyl chloride (C1-R1-PVC), glass (C1-R1-Glass)	Stainless steel (C1-R2-SS), glass (C1-R2-Glass)
Campaign No. 2 (C2)	Polyvinyl chloride (C2-R1-PVC), glass (C2-R1-Glass)	Stainless steel (C2-R2-SS), glass (C2-R2-Glass)
Campaign No. 3 (C3)	Brass (C3-R1-B), glass (C3-R1-Glass)	Titanium (C3-R2-Ti), glass (C3-R2-Glass)
Campaign No. 4 (C4)	Brass (C4-R1-B), glass (C4-R1-Glass)	Titanium (C4-R2-Ti), glass (C4-R2-Glass)
Campaign No. 5 (C5)	Stainless steel (C5-R1-SS), glass (C5-R1-Glass)	/
Campaign No. 6 (C6)	Titanium (C6-R1-SS), glass (C6-R1-Glass)	/

Except for C5 and C6, each campaign was performed with two successive runs used with the same surface water and named R1 and R2.

The glass slide coupons were used as a reference material to evaluate the variability between campaigns and assays. Subsequently, in addition to the tested material (polyvinyl chloride, stainless steel, brass, and titanium), several coupons made of glass were installed in the reactor.

The reactor was fed with Loire River freshwater (Dampierre-en-Burly, France), collected 1–3 weeks before each experiment. The water was stored in darkness in a agitated and refrigerated (4°C) tank for the duration of the experiments. Microbial and physicochemical characteristics of the inlet water are shown in Table 2. Apart from the parameters of dried suspended solids and nitrate, it can be considered that no drastic variation in water quality was observed.

**Table 2:** Microbial and physicochemical characteristics of the inlet freshwater.

		**Campaign names with date of collection**					
**Parameters**	**Unit**	**C1**	**C2**	**C3**	**C4**	**C5**	**C6**
		**7 February 2011**	**19 April 19, 2011**	**26 September 2011**	**7 March 2012**	**15 June 2011**	**6 December 2011**
Thermophilic FLA	Cells × L^−1^	< 1.05 × 10^2a^	< 1.05 × 10^2a^	< 1.05 × 10^2a^	< 1.05 × 10^2a^	ND	ND
*N. fowleri*	Cells × L^−1^	< 1.05 × 10^2a^	< 1.05 × 10^2a^	< 1.05 × 10^2a^	< 1.05 × 10^2a^		
Bacteria	Cells × L^−1^	1.5 × 10^8^	2.3 × 10^8^	1.5 × 10^8^	1.8 × 10^8^		
pH	–	8.2	8.3	8.7	8.4		
Conductivity	μS × cm^−1^	237	258	291	278		
DSS	mg × L^−1^	11	6	< 2^a^	9		
TOC	mg × L^−1^	2.9	3.3	2.7	3.7		
DOC	mg × L^−1^	2.8	2.9	2.7	3.2		
Calcium	mg × L^−1^	39	35	34	37		
Magnesium	mg × L^−1^	5.5	6	6	5.6		
Nitrate	mg × L^−1^	15	4.7	3.7	4.1		
Nitrite	mg × L^−1^	0.05	0.09	< 0.03^a^	< 0.03^a^		
Ammonium	mg × L^−1^	< 0.05^a^	0.08	< 0.05^a^	< 0.05^a^		
Copper	μg × L^−1^	< 5^a^	< 5^a^	< 5^a^	< 5^a^		

a Detection limits of the analytical methods.

The freshwater was collected between February 2011 and March 2012. FLA, free living amoebae; DSS, dried suspended solids; TOC, total organic carbon; DOC, dissolved organic carbon, ND, not determined. Refer to Table 1 for the assignment of campaign name to tested materials.

A suspension of *N. fowleri* in the trophozoite form (10^8^-10^9^ amoebic trophozoites L^−1^) was prepared extemporaneously and was inoculated into the reactor in a single injection 24 h after its start-up to reach 10^5^ trophozoites L^−1^ (final concentration). A previous study had established that these conditions allowed viable *N. fowleri* to colonize biofilms (Goudot et al., 2012).

For each assay, seven to nine samples of three coupons were randomly collected for analysis over the course of the 45 days of the experiments. The coupons were gently washed with 10 ml of bacteria-free PBS to remove cells and deposits not strongly attached to the support material (i.e., not considered part of the biofilm).

The apparent specific growth rate (μ_expo_) for amoebae and the bacteria/amoebae ratio (BAR) were calculated as previously described by Goudot et al. (2012) when data permitted. BARs were calculated for each experiment according to the **Equation (1)**:


(1) $$\begin{array}{l} BAR=\;\frac{\left(bacterial\;density\right)}{\left(amoebae\;density\right)}, \end{array}$$

where “bacteria density” and “amoebae density” are initial cell densities (cells × cm^−2^), i.e., densities measured on the substratum at the time of the injection of *N. fowleri*. Therefore, BAR is assumed to represent the amount of prey available *per* amoeba in the biofilm at the time of the amoeba injection into the reactor.

### 2.5 Statistical analysis

Comparisons between samples (assays) were assessed using ANOVA, where statistical significance was considered (*p* < 0.05). Statistical analyses were performed using XLSTAT Version 2010.1.01 (Addinsoft, Paris, France).

Error values for the fitting curves were given by Kaleidagraph software v.5.0.

## 3 Results

### 3.1 Characteristics of the biofilms

**Figure 2:**
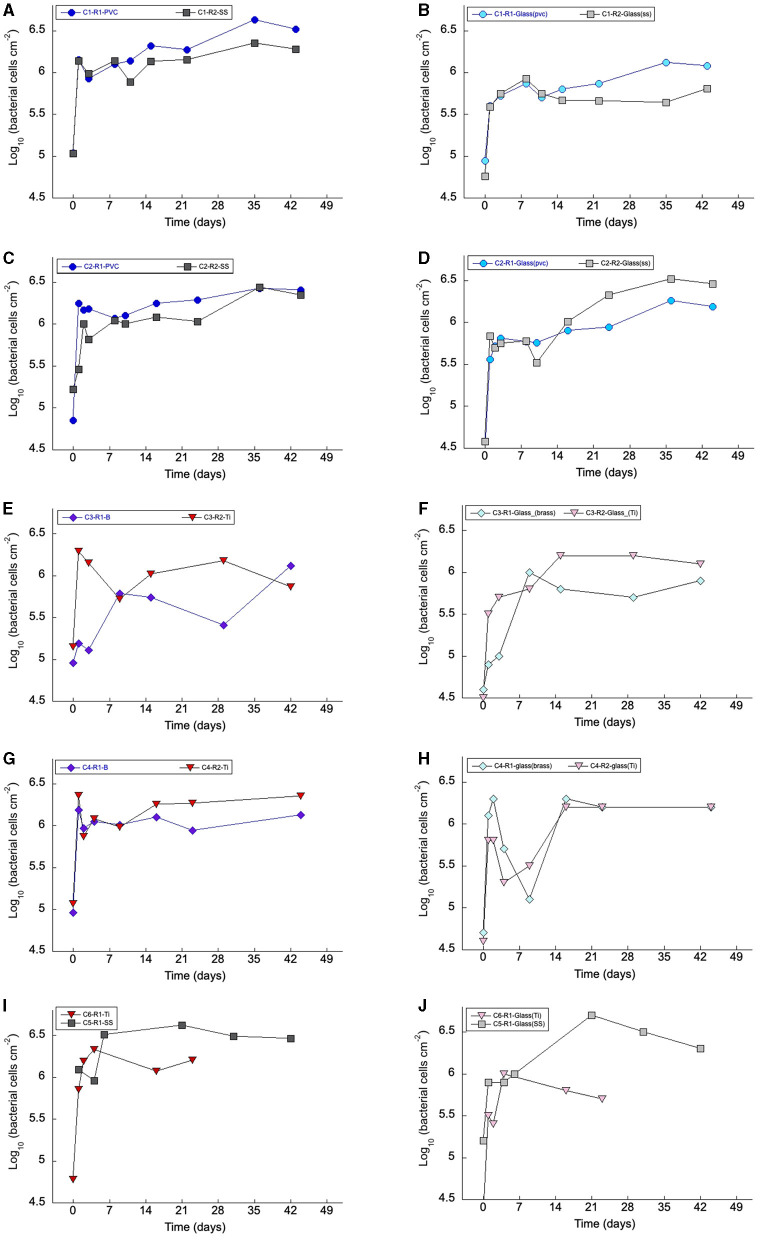
Temporal fluctuations of the bacterial biofilm densities under 42°C for all assays. **(A)**, C1-R1-PVC (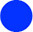) and C1-R2-SS (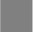); **(B)**, C1-R1-Glass (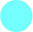) and C1-R2-Glass (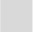); **(C)**, C2-R1-PVC (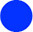) and C2-R2-SS (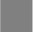); **(D)** C2-R1-Glass (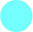) and C2-R2-Glass (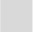); **(E)**, C3-R1-B (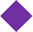) and C3-R2-Ti (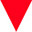); **(F)**, C3-R1-Glass (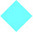) and C3-R2-Glass (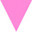); **(G)** C4-R1-B (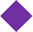) and C4-R2-Ti (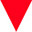); **(H)**, C4-R1-Glass (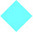) and C4-R2-glass (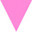); **(I)**, C6-R1-Ti (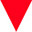) and C5-R1-SS (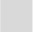); **(J)**, C6-R1-Glass (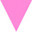) and C5-R1-Glass (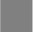). C1, C2, C3, C4, C5, and C6 are the name of the campaigns, each including two runs named R1 and R2. See Table 1 for assignments. PCV, polyvinyl chloride; SS, stainless steel; Ti, titanium; B, brass.

The variation in bacterial cell density in the biofilms developed on the tested materials (polyvinyl chloride, stainless steel, brass, and titanium) was monitored over time (Figure 2). The first two campaigns, C1 and C2, were performed with polyvinyl chloride and stainless steel (Figures 2A, C), and campaigns C3 and C4 were performed with titanium and brass (Figures 2E, G). Two additional campaigns, C5 and C6, were performed on stainless steel and titanium (Figure 2I). In addition, experiments involving the biofilms growing on glass coupons, which shared the same reactor as tested materials serving as reference material, were conducted (Figures 2B, D, F, H, J).

The results show that all substrata, including glass coupons, were colonized by bacteria (Figure 2). This colonization is assumed to be a result of both the attachment and growth of the bacteria. A temporal analysis of the bacterial colonization kinetics reveals two major phases: a first phase from 0 to 3 days characterized by a fast increase in bacterial densities, followed by a second phase from 3 to 45 days characterized by a slowdown or slowup in biofilm development. A more pronounced decrease, than in the other tests, occurred with the glass coupons of the C4 campaign, where brass or titanium was tested (runs R1 and R2) between 2 and 16 days (Figure 2H). However, on a statistical basis, the results did not allow for the establishment of a ranking list of the tested materials according to their ability to promote or not the bacterial colonization, since no significant difference was noted between the assays. This aspect suggests that the biofilm implantation conditions of the autochthonous amoebae (FLA) and *N. fowleri* we conducted were similar between the materials.

### 3.2 Dynamic of the indigenous amoebae and *Naegleria fowleri* in the biofilm

**Figure 3:**
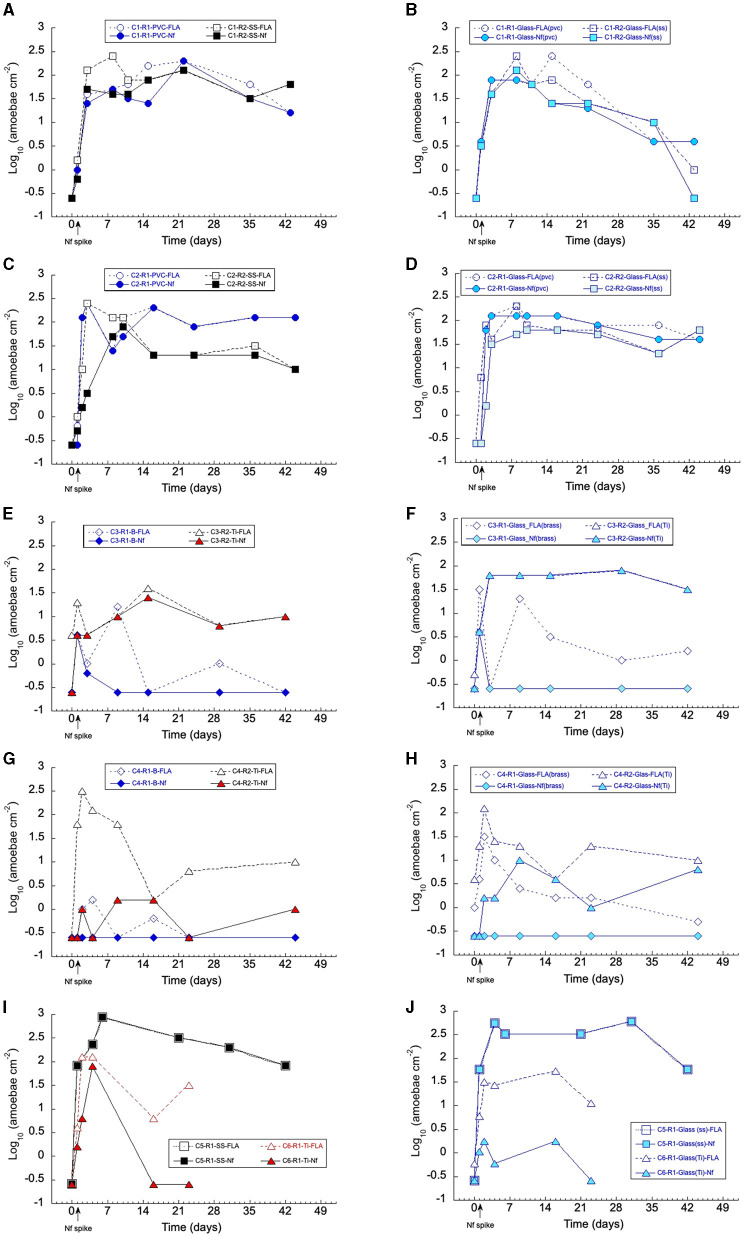
Temporal fluctuations of the amoebae in the biofilms under 42°C for campaigns C1–C6: **(A, C)** on *PVC* = polyvinyl chloride (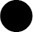,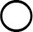) and *SS* = stainless steel (■, □); **(E, G)** on *B* = brass (♦, ♢) and *Ti* = titanium (▴, ▵); **(I)**, on *SS* and *Ti*; **(B, D)**, on glass (with *PVC* or *SS* coupons); **(F, H)**, on glass (with *B* or *Ti*); **(J)** on glass (with *SS* or *Ti*). Dashed line and open symbols = thermophilic free-living amoebae (FLA); solid line and closed symbol = *Naegleria fowleri*. C1, C2, C3, C4, C5, and C6 are the name of the campaigns, each including two runs named R1 and R2. See Table 1 for assignments. The arrow indicates when the spike of *N. fowleri* was applied into the reactors.

In addition to bacteria, thermophilic FLA and *N. fowleri* densities were monitored over the course of the experiments (Figure 3). In all cases, no thermophilic FLA was detected on the substrata (<0.3 amoeba cm^−2^) before the spike of *N. fowleri* and in the fresh water inlet (Table 2).

For campaigns C1, C2, and C5 all done on PVC and SS, a significant increase of *N. fowleri* within a range of 30–60 *N. fowleri* cm^−2^ was shown on SS and PVC coupons within the first weeks after the injection (Figures 3A, C, I). These results clearly demonstrate that *N. fowleri* has colonized the biofilm of the coupons. After reaching 100–250 cells × cm^−2^, a slight decrease in the amoebae density was observed, followed by a relative stability or a small decrease, indicating that the amoebae population was maintained on these materials during the 40 days of the experiment (Figures 3A, C, I). Thus, whatever the two materials investigated (PVC and SS), the colonization of the coupons by amoebae was not significantly different. Actually, the colonization was mainly due to *N. fowleri*, which remains the main detectable thermophilic FLA (compare FLA with *N. fowleri*). Similar trends were obtained with glass sharing the same reactor (Figures 3B, D, J), although a relative stronger decline in amoebae was observed with glass for the two runs of the C1 campaign (Figure 3B). The amoebal surface colonization was also coupled with the persistence of autochthonous amoebae as well as the experimentally injected *N. fowleri* (Supplementary Figure S1) in water over the 6 weeks of assays.

With the C3, C4, and C6 campaigns all done on brass and titanium, the amoeba colonization profile was different. On brass, the colonization of the biofilm was limited to 15 or 2 FLA cells cm^−2^, and *N. fowleri* was not able to colonize the biofilm after the spike (<0.3 cells cm^−2^ after the 7th day, Figures 3E, G). The glass coupons sharing the same reactor with brass coupons (C3-R1) indicated a similar trend, namely, a transient installation of FLA with no true colonization by *N. fowleri*, which was no longer detectable after 2 days (Figures 3F, H). In contrast, runs with titanium showed a significant increase in FLA during the first days, with a maximum of 40–300 amoebae cm^−2^, depending on the campaigns (Figures 3E, G, I). Thereafter, the FLA densities were relatively stabilized ~10–30 amoebae cm^−2^. Moreover, on titanium, the FLA presented equal or higher densities than those of *N. fowleri* (campaigns C4-R2 and C6-R1), suggesting the emergence of autochthonous amoebae at the expense of *N. fowleri* (Figures 3G, I). Based on morphological characteristics (not shown), such autochthonous amoebae were assumed to belong to the genus *Hartmannella*. Compared to other assays in the present study, this appears to be the fastest and highest implantation of indigenous amoebae. A decrease to a range of 1–10 amoebae cm^−2^ of this FLA population was observed from the third day. Similar colonization of FLA and *N. fowleri* was noted on the reference material (glass) (Figures 3F, H, J). These differences in the surface colonization of amoebae between brass and titanium were also observed with the same trends in water (Supplementary Figure S1) during the 6 weeks of testing.

Finally, these results suggest that the implantation and growth of amoebae, FLA, and *N. fowleri* were favored in the freshwater biofilms developed on polyvinyl chloride, stainless steel, or, to a lesser extent, titanium, contrary to brass, which led to the disappearance of amoebae and *N. fowleri* in particular.

### 3.3 Trophic relationships between bacteria and *Naegleria fowleri*

**Figure 4:**
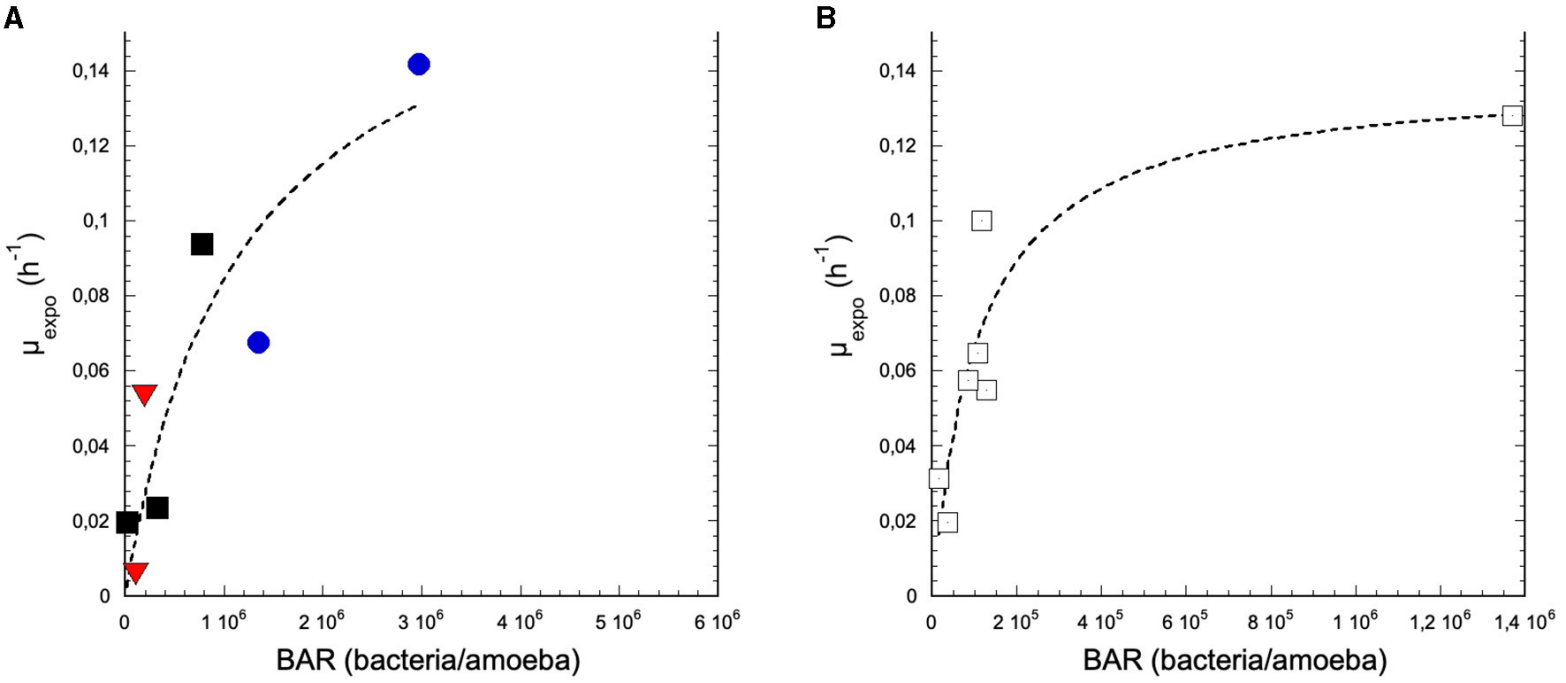
Variation of the apparent specific growth rate (μ_expo_) as a function of the bacteria/amoeba ratio (BAR) (*N. fowleri*) at 42°C. The assays considered are **(A)** C1-R1-PVC and C2-R1-PVC (°); C1-R2-SS, C2-R2-SS, and C5-R1-SS (■); C3-R2-Ti and C6-R1-Ti (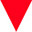); **(B)** glass coupons assays sharing the same reactor as other coupons in PVC, SS, or Ti. C1, C2, C3, C5, and C6 are the names of the campaigns, each including two runs named R1 and R2. See Table 1 for assignments. The data were adjusted with the Michaelis-Menten relation (*R* = 0.934). PCV, polyvinyl chloride; SS, stainless steel; and Ti, titanium.

For runs where *N. fowleri* growth was observed, apparent specific growth rates (μ_expo_) were calculated and plotted against the bacteria/amoeba ratio (**Equation 1**; Figure 4). The runs C3-R1-B, C4-R1-B, and C4-R2-T could not be considered due to the lack of growth of *N. fowleri*.

The increase of μ_expo_ with *BAR* was adjusted by the Monod equation (**Equation 2**):


(2) $$\begin{array}{l} \mu_{expo}=\;\frac{\left(\mu_{\max}\times BAR\right)}{\left(K_{s}+BAR\right)} \end{array}$$

In such an equation, we assume that *BAR* is equivalent to the growth-limiting substrate concentration (Goudot et al., 2012). The maximal specific growth rate (μ_max_) derived from the **Equation (2)** is equal to 0.18 ± 0.07 h^−1^ for all tested materials (PVC, SS, and T) and 0.14 ± 0.02 h^−1^ for glass. The half saturation constants (*K*_S_) were 1.2 × 10^6^ ± 0.9 × 10^6^ bacteria × amoeba^−1^ and 1.1 × 10^5^ ± 0.5 × 10^5^ bacteria × amoeba^−1^ and the generation time (*G*) was 5 and 4 h for glass and all other materials, respectively (Figure 4). These data suggest that the variability of *N. fowleri* observed on glass, PVC, SS, and Ti was explained by the variation in the available substrates (i.e., biofilm cell density per amoeba, or *BAR*). Since *N. fowleri* colonization was not observed on any of the brass coupons and the apparent density of biofilm bacteria was not significantly different from that of the other substrata (Figures 2E, G), one can consider that *BAR* was not the limiting growth factor on brass and on glass sharing the same reactor as brass coupons.

### 3.4 Copper level in biofilm and water bulk

In our experiments, the measurement of the dissolved copper in the water for all the assays showed that in the presence of brass coupons (campaigns C3 and C4), the concentration of dissolved copper (30–110 μg L^−1^) was 10–30 times greater than the copper concentration in water in contact with other materials (under the detection limit of 3 μg L^−1^) (not shown). To check the presence of copper within biofilms, analyses were conducted on biofilm extracts from campaigns C3 and C4. All extracts other than those from brass showed very low or values below the detection limit (<0.1 μg mg^−1^ dry weight, DW). On the contrary, biofilms developed onto brass coupons presented an average copper concentration of 9 ± 2 or 17 ± 5 μg mg^−1^ DW (Supplementary Table S1).

## 4 Discussion

The number of bacterial prey provided by the biofilm was proposed as one of the primary factors controlling the growth of *N. fowleri* (Goudot et al., 2012). Temperature, the system hydraulics, and the water quality are other factors influencing the growth of FLA (Lam et al., 2019; Stahl and Olson, 2021). They were controlled and relatively uniform for the assays of each run. Therefore, they can be discounted as influencing the amoebae colonization. The material substrate is another relevant factor that was examined in the present study. This substrate could directly or indirectly influence the growth of amoebae by acting on the amoebae itself (e.g., diffusion of toxic substances) or by acting on the amoeba preys (i.e., by affecting the availability of preys in the biofilm), respectively.

### 4.1 Indirect influence of the substratum on amoebae growth

#### 4.1.1 Bacterial cell density

In the present study, the bacterial colonization rates on various tested materials were in the same order of magnitude as in other previous studies, that is, ~10^6^ cells cm^−2^ on polyvinyl chloride or stainless steel (Jang, 2011; Simões et al., 2012; Papciak et al., 2019). We can reasonably expect that bacterial colonization on brass to be significantly lower than for other materials because the copper, present in brass (~70% in mass), could exhibit toxic effects. Indeed, the literature reports studies mentioning the negative effects of brass (or copper as well) on the development of biofilm or bacterial strains of major clinical interest (Yu et al., 2010; Grass et al., 2011; Krishnan et al., 2017; Gomes et al., 2019; Pontin et al., 2021). Nevertheless, with environments exhibiting diversified bacterial communities, such as river water or drinking water, there is no clear consensus, and stainless steel, rather than brass or copper, can also be a material associated with the lowest bacterial density (Lehtola et al., 2004; Hyun et al., 2011; Waines et al., 2011; Ginige et al., 2017; Inkinen et al., 2018). The same observation can be made with titanium; if some studies indicate a lower bacterial colonization of titanium, others indicate no notable differences (Zoubos et al., 2012). Thus, the influence of these materials on the bacterial density of the colonizing biofilm is not clearly established.

To quantify the influence of cell density on amoebae growth, and especially *N. fowleri*, the BAR parameter (**Equation 1**) has been previously suggested. This ratio was exhibited to control the colonization of *N. fowleri* in biofilms on the glass material (Goudot et al., 2012). In this present study, the relationship between μ_expo_ and BAR, regardless of the material (except brass because no significant growth of amoebae was observed), indicated values for μ_max_ and *K*_s_ (0.18 h^−1^ and 1.2 × 10^6^ bacteria/amoeba and 0.14 h^−1^ and 1.1 × 10^5^ bacteria/amoeba, on various materials and glass, respectively) relatively close to those found on glass under the same operational conditions (0.23 h^−1^ and 1.2 × 10^5^ bacteria/amoeba) (Goudot et al., 2012). Consequently, nutritional availability, represented by the BAR parameter, would more strongly influence the colonization of the biofilm by *N. fowleri* rather than nature of the material among those tested (PVC, SS, Ti, and glass), with the exception of brass.

#### 4.1.2 Effects of copper on the biofilm community

The nature of a material constituting the substrata colonized by a biofilm has been suspected to influence bacterial diversity (Hyun et al., 2011; Lu et al., 2014; Pinto et al., 2019; Learbuch et al., 2021). Since, among all materials tested in the present study, only brass showed a significant effect on amoebae colonization, the effect of copper constituting the brass is expected. Indeed, this phenomenon has already been demonstrated by Fuma et al. (2003), who have evaluated the effects of copper on a microcosm consisting of populations of the flagellate alga *Euglena gracilis* as a producer, the ciliate protozoan *Tetrahymena thermophila* as a consumer and the bacterium *Escherichia coli* as a decomposer. The results showed that 6.35 mg L^−1^ of copper extinguished first *E. coli* and then *T. thermophila* in the microcosm. No action was noted for the flagellate alga *E. gracilis*. The authors considered the extinction of *E. coli* to be a direct effect of copper, because 6.35 mg/L of copper almost extinguished *E. coli* in a pure culture. In contrast, they considered the extinction of *T. thermophila* in the microcosm to be a community-level effect because 6.35 mg L^−1^ did not affect the cell density of *T. thermophila* in the pure-culture system. Under other conditions, copper was shown to have a negative effect on the biofilm bacterial community. For example, Boivin et al. (2006) showed that exposure to 432 μg L^−1^ of copper provoked distinct changes in the DGGE profiles of the bacterial consortia, which did not reverse upon copper depuration, in environmental biofilms. Zhang et al. (2006) reported that in wastewater biofilms, copper contamination was elected for specific species that were able to tolerate this stress and that may contribute to its remediation. Therefore, we cannot exclude that the relatively low concentration of copper we measured in the water bulk of the reactor (10–110 μg L^−1^) exerts an effect on the bacterial structure community in the biofilm.

Amoebae appear to tolerate higher copper concentrations than bacteria, and according to previous work by Lu et al. (2014), this could provide an advantage to amoeba-resistant bacteria (e.g., *Legionella pneumophila*), which appeared more abundant in biofilms downstream of copper materials; the hypothesis of a benefit due to the presence of their copper-resistant amoebic host has therefore been suggested (Lu et al., 2014). In the same vein, we can speculate that a significant development of these ARBs would ultimately affect their amoebic hosts. This fact could explain in our results, why the amoebae from the glass coupons that shared the same reactor as the brass coupons did not develop either.

### 4.2 Direct influence of substratum on FLA and *N. fowleri*

No significant colonization by the amoebae was observed on brass, suggesting that brass does not promote an effective colonization. It is then logical to consider a direct toxic effect of copper. Indeed, while small amounts of copper are an essential trace element in most pro- and eukaryotic organisms, it can become toxic with higher concentrations. This fact has already been reported that electrolytically generated copper and silver concentrations at a ratio up to 80:800 μg L^−1^, respectively, and a dissolved copper concentration of 500 μg L^−1^ caused no significant inactivation of *N. fowleri* after 72 h exposure (Cassells et al., 1995). More recently, Grechnikova et al. (2020) found a maximum semi-inhibitory concentration (IC_50_) for *N. fowleri* equal to 1.6 mM (100 mg L^−1^). This fact suggests that this amoeba a relatively copper-resistant eukaryotic microorganism and copper concentrations in the water of our reactor (1,000 × lower than the IC_50_ mentioned above) are unlikely to directly affect *N. fowleri*. However, the copper level extracted from the biofilm on brass coupons (9–17 μg mg^−1^ DW) suggests that the copper concentration present in the biofilm could locally reach toxic values for amoebae: 9–17 μg mg^−1^ DW corresponds to ~8 μg cm^−2^ (Supplementary Table S1, taking into account the value of DW for three coupons of 22 cm^2^ each), and by considering a biofilm thick of 20 μm (i.e., a final volume of biofilm of ~2 mm^3^ cm^−2)^, it would mean amoebae eat biofilms having ~4 mg Cu cm^−3^ (i.e., an immense 4 g L^−1^). Therefore, we cannot exclude a direct toxic effect of copper on amoebae. However, the concentrations for which such an effect is expected should also influence the bacteria present in the biofilms. However, our results do not show anything of the sort. To ensure this aspect, further research into the levels of copper actually absorbed by amoebae living on copper substratum should be conducted.

### 4.3 Competition between amoebae

Competition between amoebae is another parameter that can control biofilm colonization by *N. fowleri*, as has already been discussed in other studies (Stahl and Olson, 2021). In the present study, the runs showing a higher density of FLA than that of *N. fowleri* suggest that native amoebae took over the established *N. fowleri*. However, tests with a similar or slightly lower *N. fowleri* density than FLA indicated that *N. fowleri* had successfully implanted itself. In the first cases, this could result from competition favorable to the autochthonous; in the second case, the competition would have been favorable to *N. fowleri*. We can therefore consider that small heterogeneities in the densities of native amoebae present in the water of the rivers supplying the reactors could have contributed to the differences obtained in our study, rather than a different behavior of the amoebae with regard to the nature of the substrate. To draw a conclusive statement on this point, the ideal would have been to carry out all the materials to be tested simultaneously with the same river water.

Finally, after examination of these hypotheses, the direct or indirect inhibition of *N. fowleri* by the direct toxic effect of copper or by trophic relationship with bacteria (prey-predator) remains realistic. It would then be interesting to observe the variation in diversity of the bacterial communities when they are exposed to brass because this could help us identify, if any, possible bacterial factors governing the abundance of *N. fowleri*. However, this possibility remains to be confirmed. It is conceivable that in direct contact with brass, the toxicity threshold is reached or even exceeded (i.e., more than a factor of a thousand, as our estimation of the copper level suggests). Therefore, the copper level within the biofilm, would be much greater than that found in the water bulk and would be becomes sufficient to inhibit the growth of *N. fowleri*.

## 5 Conclusion

This work highlights that (1) experimentally doped *N. fowleri* is equally able to colonize polyvinyl chloride, stainless steel, or titanium but not brass; (2) BAR representing nutritional availability for FLA appears to be the driving force behind biofilm colonization by *N. fowleri* and FLA in the biofilm rather than in the materials tested (with the exception of brass); (3) the generation time of *N. fowleri* in the biofilm was estimated at 4 h (with the exception of brass); and (4) FLA colonization is inhibited by a direct or indirect effect of copper, which needs to be studied further.

## Data availability statement

The raw data supporting the conclusions of this article will be made available by the authors, without undue reservation.

## Author contributions

SG: Conceptualization, Data curation, Formal analysis, Investigation, Methodology, Writing – original draft. LM: Conceptualization, Supervision, Writing – review & editing. PH: Conceptualization, Funding acquisition, Project administration, Supervision, Validation, Writing – review & editing. SS: Conceptualization, Project administration, Writing – review & editing. FJ: Conceptualization, Funding acquisition, Supervision, Validation, Writing – review & editing.
