# Supplementary material for: Growth dynamic of biofilm-associated Naegleria fowleri in freshwater on various materials
Source: Front Microbiol. 2024 Mar 6;15:1369665. doi: 10.3389/fmicb.2024.1369665 (PMC10951111; doi:10.3389/fmicb.2024.1369665)
Supplement: Supplementary file 1 [file Data_Sheet_1.docx]

Supplementary Material

Growth dynamic of biofilm-associated *Naegleria fowleri* in freshwater on various materials

Sébastien Goudot, Laurence Mathieu, Pascaline Herbelin, Sylvie Soreau, Frédéric P.A. Jorand*

*** Correspondence:** Corresponding Author: [frederic.jorand@univ-lorraine.fr](mailto:frederic.jorand@univ-lorraine.fr)

# Supplementary Figures and Tables

## Supplementary Figures

**Figure S1.** Cell densities of *Naegleria fowleri* (Nf, squares and circles) and other free living amoebae (FLA, crosses) as a function of time (days) in the bulk water of the reactor runs for various substrata: A, B, polyvinyl chloride and glass (C1R1 and C2R1 campaigns, respectively); C, D, stainless steel and glass (C1R2, and C2R2 with C5R1 campaigns, respectively); E, F, brass and glass (C3R1 and C4R1 campaigns, respectively); G, H, titanium and glass (C3R2 and C4R2 with C6R1, respectively), see Table 1 for assignment. Note the detection limit for amoebae was 10^2^ cells L^-1^. The arrows indicate when the spike of *N. fowleri* suspension was done.

|  |  |
| --- | --- |
|  |  |
|  |  |
|   Supplementary Figure S1 |  |

Table S1. Physico-chemicals data from biofilms extracts. Analyses have been performed on biofilm extracted from three coupons and pooled, at days 1 and 16 (n = 2) or at days 1, 9 and 29 (n = 3). DW = dry weight. See Table 1 for assignment of assays names.

| **Assays** | **Material of the coupons** | **DW**  **(mg mL^-1^)** | **Cu**  **(µg mg^-1^ DW)** |
| --- | --- | --- | --- |
| C3-R1-B | Brass | 0.38 ± 0.04 (n = 3) | 9 ± 2 (n = 3) |
| C3-R1-Glass | Glass | 0.34 ± 0.09 (n = 3) | < LD (n = 3) |
| C3-R2-Ti | Titanium | 0.31 ± 0.13 (n = 3) | 0.12 ± 0.03 (n = 3) |
| C3-R2-Glass | Glass | 0.32 ± 0.09 (n = 3) | < LD (n = 3) |
| C4-R1-B | Brass | 0.20 ± 0.01 (n = 2) | 17 ± 5 (n = 2) |
| C4-R1-Glass | Glass | 0.12 ± 0.01 (n = 2) | 0.3 ± 0.3 (n = 2) |
| C4-R2-Ti | Titanium | 0.10 ± 0.01 (n = 2) | 1.2 ± 0.8 (n = 2) |
| C4-R2-Glass | Glass | 0.12 ± 0.01 (n = 2) | < LD (n = 2) |
